# The mediating effects of self-concept on the relationship between parenting styles and young children’s social problem-solving in Türkiye

**DOI:** 10.3389/fpsyg.2025.1444648

**Published:** 2025-02-05

**Authors:** Ahmet Ayık, Türker Sezer, Sinan Koçyiğit

**Affiliations:** ^1^Department of Educational Sciences, Kazım Karabekir Education Faculty, Atatürk University, Erzurum, Türkiye; ^2^Department of Preschool Teacher Education, Education Faculty, Bolu Abant Izzet Baysal University, Bolu, Türkiye; ^3^Department of Preschool Teacher Education, Kazım Karabekir Education Faculty, Atatürk University, Erzurum, Türkiye

**Keywords:** parenting styles, self-esteem, self-efficacy, social problem solving, 48–72-month-old-children

## Abstract

The aim of this study was to investigate the associations between parenting styles, young children’s social problem-solving skills, and the mediating role of self-concept in a sample of 200 Turkish preschoolers aged 48–72 months, with an equal distribution of male and female participants. The present study was designed using a cross-sectional survey model in order to achieve the descriptive and predictive aims of the research. Data were collected through individual sessions with the children. During these sessions, the children were administered the Wally Social Problems Test and the DeMoulin Self-Concept Development Scale, while the mothers completed the Parenting Attitudes Scale and the Demographic Information Form. The mediating role of self-esteem and self-efficacy in the relationship between parenting styles and children’s social problem-solving skills was examined using PROCESS MACRO. The results supported the proposed model, demonstrating that the impact of democratic parenting style on social problem-solving skills was partially mediated by self-concept, specifically self-esteem, as a parenting measure. These findings suggest that self-esteem is an essential individual characteristic to consider in relation to preschoolers’ social relationships, in addition to the influence of democratic parenting style behaviors.

## 1 Introduction

Social Problem Solving (SPS) refers to the cognitive-emotional-behavioral process that enables individuals to identify and use effective coping strategies for specific problem situations encountered in daily life. It is related to establishing social interaction and adapting to the social environment, which is crucial for leading a healthy life. SPS has been extensively researched over the past 50 years (Chang et al., 2020; D'zurilla and Goldfried, 1971). The lack of effective, positive, and diverse strategies for social problems in the social environment of children is considered an important developmental risk factor (Rubin and Rose-Krasnor, 1992). SPS are considered key developmental skills for children’s social, and emotional adaptation (Ziv, 2013). Children with SPS are not isolated from their peers and have higher acceptance among peers (Paswan et al., 2014). High acceptance supports school adjustment, and increases academic achievement (Dubow et al., 1991). On the contrary, low levels of SPS are associated with behavioral (Malik et al., 2006), and psychological problems (Chang et al., 2020).

Researchers conducted in the past 50 years has consistently found a connection between parenting and SPS. Factors such as exposure to aggression, restrictive discipline (Pettit et al., 1988), maternal sensitivity, attachment (Raikes and Thompson, 2008), maternal acceptance-rejection (Tepeli and Yilmaz, 2013), positive parenting, and socio-demographic variables have been shown to impact a child’s SPS (Su et al., 2020). From the research findings it can be concluded that negative family experiences, such as harsh discipline, low acceptance, insensitivity to the child’s needs and insecure attachment, have a detrimental effect on children’s SPS. Conversely, positive family experiences, including warmth, sensitivity and parental guidance, have a positive effect on children’s SPS.

Research has shown that both family dynamics and child characteristics have direct and interactive effects on self-concept, similar to SPS (Brown et al., 2009). Meta-analyses and cross-cultural studies have found that the immediate social environment of the child, including peers, teachers, and family, has a mutual and continuous influence on self-concept, with the family playing the most important role (Brown et al., 2009; Harris and Orth, 2020). Studies have provided evidence that parental sensitivity to the interests and needs of their children has positive effects on their self-concept (Paulus et al., 2018), while neglect, low acceptance, and high expectations have negative effects (Smith, 2007).

Both SPS and self-concept play a significant role in an individual’s adaptation to their environment, and since they are both affected by parent–child interactions, they are closely interrelated. These two concepts are particularly relevant in the context of behavior problems (D'zurilla et al., 2003) and peer relationships in young children (Öneren Şendil and Erden, 2014). Previous studies that directly investigated the relationship between SPS and self-concept have supported the idea that they are interconnected (D'zurilla et al., 2003; Verschueren et al., 2012). Family experiences, in turn, can significantly impact both SPS and self-concept. Therefore, the aim of this study is to investigate whether self-concept (self-esteem and self-efficacy) plays a mediating role between parenting styles and SPS in young children. As children transition from their family environment to preschool, which is their first significant social environment, they begin to reflect on their parental experiences (Amerijckx and Humblet, 2015). Developing effective problem-solving skills (Petersen, 2012; cited in Appl et al., 2017) is crucial to helping children adapt to this new social structure and succeed in learning tasks. Preschool children with high SPS levels demonstrate a greater ability to adapt to their peers (Paswan et al., 2014) and teachers (Weyns et al., 2019), which in turn, supports academic achievement (Walker and Henderson, 2012). Additionally, children who demonstrate high SPS levels in the preschool period tend to maintain this trend in primary school and beyond (Coulombe and Yates, 2018). Therefore, this study aims to identify the variables that impact SPS during the early childhood period.

### 1.1 Conceptual framework

There are four major perspectives on the theoretical basis of social problem-solving (SPS) falling within the scope of social competence. These include a multidimensional problem-solving skill process (Goldfried and D'Zurilla, 1969), Interpersonal Cognitive Problem-Solving Skills (ICPS) (Spivack and Shure, 1974), the Social Information Processing Model (SIP) (Rubin and Krasnor, 1986), and the social-cognitive based SIP model (Dodge, 1986). All models highlight that solving social problems is a process requiring a series of steps and influenced by cognitive development, experiences, environmental factors, emotions, and self-perception. In this study, SPS data was collected using a measurement tool based on the ICPS and SIP models (Tepeli and Yilmaz, 2013).

The study of parenting styles and practices has long been of interest to social scientists (see, e.g., Belsky, 1984; Baumrind, 1967). Studies on parenting have addressed different dimensions of this concept. For example, Belsky (1984) presented a model in which he argued that parenting has a multidimensional structure and that parenting is influenced by the characteristics of the parent (e.g., personality and psychological functioning), the characteristics of the child (e.g., temperament) and the characteristics of the social context of the family (e.g., socio-economic structure). In addition, Rohner (1986) developed the Acceptance-Rejection theory on the basis of parents’ rejection of their children. In the theory, parents are evaluated in the dimensions of coldness and lack of affection (warmth and love), aggression and hatred, indifference and neglect and undifferentiated rejection (Rohner, 1986; Rohner and Khaleque, 2002). In addition to these, it is seen that parenting is addressed with the dimensions of sensitivity (e.g., accessibility, acceptance, cooperation, communication, awareness, warmth) and responsiveness (e.g., providing guidance and support, self-efficacy and safety) (Bornstein, 1989). In other studies, Barber (1996) and Steinberg (2005) examined the psychological control dimension (behaviors towards children’s feelings and thoughts) applied by parents. Especially in the 1970s and 1980s, we also see the typologies approach to parenting behaviors proposed by Baumrind (1971) and Maccoby and Martin (1983). Parenting typologies are differentiated according to the interest/acceptance and demanding behaviors of parents towards their children (Baumrind, 1991, 2005). Baumrind (1967, 1971, 1991) defined three basic parenting typologies: democratic/balanced, authoritative and permissive. Maccoby and Martin (1983) approached Baumrind’s conceptualization from a new perspective and explained parenting as four typologies within the vertical relationship of the interest/acceptance and demand dimensions. According to this arrangement, parents are categorized as authoritative (high demand and acceptance), authoritarian (high demand, low acceptance), permissive (low demand, high acceptance) and neglectful (low demand, low acceptance).

Thomasgard et al. (1995) defined parenting practices as a continuum of control from neglect to overprotection and associated overprotection with characteristics such as excessive control and supervision, separation problems, dependence and control according to the developmental level of the child as in authoritarian parenting. Overprotective parenting, motivated by anxiety and harm prevention (Brussoni and Olsen, 2012; Brenning et al., 2017), limits children’s autonomy through excessive monitoring and control, prioritizing safety over emotional connection. Conversely, authoritarian parenting, which focuses on strict discipline and control (Baumrind, 1991; Suarez-Morales and Torres, 2021), inhibits autonomy through the enforcement of rigid rules and the cultivation of hierarchical and emotionally detached relations (Brenning et al., 2017). For this study, the neglectful style was replaced with the overprotective style, which involves parents not supporting the child’s individuality and doing tasks that the child can do on their own (Karabulut-Demir and Şendil, 2008).

Self-concept is a complex construct that describes an individual’s perception and evaluation of their own mental, physical, and social functional status. It has a dynamic and reciprocal relationship with behavior and is characterized by a hierarchical, stable, evaluative, and differentiated structure (Ling et al., 2013; Shavelson et al., 1976). This study examines two sub-dimensions of self-concept, namely self-esteem and self-efficacy. Self-esteem refers to a person’s positive or negative attitude towards themselves (Rosenberg et al., 1995), whereas self-efficacy refers to the belief in one’s ability to organize and execute actions necessary to manage prospective situations (Bandura, 1997, p. 3). This study considers the conceptual framework of self-concept based on sensitivity to school (self-efficacy) and sensitivity towards self (self-esteem), as proposed by Demoulin (1998).

### 1.2 Relationship between SPS and parenting

The development of SPS begins early in a child’s life through family interactions and is heavily influenced by parents (Mott and Krane, 1994). Children learn problem-solving strategies by observing how their parents handle similar situations (Pettit et al., 1988). Parenting styles have been found to be closely associated with children’s social skills, behavioral problems, and overall well-being. Research has shown that preschool children with democratic/authoritative parents exhibit higher levels of social competence, and positive peer relationships, as well as increased self-confidence and emotional regulation abilities, compared to children with permissive or authoritarian parents (Baumrind, 1971). Recent studies support these findings, showing that democratic parenting style reduces behavior problems in children, while permissive, authoritarian, and neglectful/uninvolved parenting styles increase such problems (Eisenberg et al., 2009; Stülb et al., 2019). Additionally, overprotective parenting style has been linked to socialization problems, exclusion from peers, anxiety, and fear behaviors in children (Zhu et al., 2021). Thus, parenting styles remain one of the most critical factors influencing children’s social competence. In summary, effective parenting has a direct and positive impact on children’s social behaviors, while dysfunctional parenting is linked to children’s aggressive and introverted behaviors (Kim and Shin, 2020).

According to the SPS perspective, children who possess high social competence and exhibit socially acceptable behaviors (Kim and Shin, 2020) are more likely to utilize effective problem-solving strategies when faced with interpersonal conflicts, in contrast to their counterparts who exhibit behavioral problems and low social competence and struggle to employ appropriate strategies (Baumrind, 1971; Rudolph and Heller, 1997). Building upon these findings, we hypothesized that parenting styles have a significant impact on the development of SPS in children.

### 1.3 Relationship between parenting and self-concept

Regarding attachment, children develop beliefs about their own selves based on the sensitivity and sensibility of their caregivers. These beliefs become central to their personalities functioning throughout life (Bowlby, 1982). Parents, the attachment figures for children from the moment they are born, continue to have psychosocial effects on their children even in adulthood (Martinez-Escudero et al., 2020). The results of the current studies examining self-esteem, which is one of the concepts in psychosocial development, provide evidence of the long-term effects generated by parents (Krauss et al., 2020; Orth, 2018).

Family dynamics and child characteristics directly and interactively affect children’s self-concept (Brown et al., 2009). According to meta-analysis and review studies, parenting styles (even though age groups are different) affect individuals’ self-esteem (Jadon and Tripathi, 2017; Pinquart and Gerke, 2019) and authoritarian and neglectful parenting had negative effects, while democratic/authoritative and tolerant parenting had positive effects (Jadon and Tripathi, 2017; Martinez et al., 2020; Pinquart and Gerke, 2019). Similar to the results about self-esteem, it was found that a democratic parenting style positively affects general self-efficacy and academic self-efficacy, but authoritarian and permissive styles are not related to self-efficacy (Masud et al., 2016; Qazi, 2009). Therefore, supportive parental behaviors were found to have positive effects on self-efficacy (Yomtov et al., 2015).

The influence of family on children’s development is a topic that requires consideration of cultural differences. In a study conducted by Rudy and Grusec (2006), it was noted that the effect of family influence on children’s behavior varies across different cultures. For example, in Chinese culture where family ties are stronger, the controlling behaviors of parents were reported to have a negative impact on children’s self-esteem and confidence (Chao, 1994). Additionally, in more collectivist cultures such as Turkish culture, it is said that the influence of family on children’s development is stronger, and the cultural values of the family play an important role in shaping children’s behaviors (Hofstede et al., 2010; Özdemir et al., 2017). However, in individualistic cultures like Western culture, where independence and autonomy are valued, the influence of family is less (Belsky, 1984). Therefore, considering cultural variables is crucial as the role of family in children’s development is related to these variables. In this context, it can be argued that a relationship exists between parenting and self-esteem and self-efficacy. Therefore, a hypothesis was developed for this relationship in the present research.

### 1.4 Self-concept as a mediator

As stated above, self-concept is affected by parent–child relationships. Some studies found associations between self-concept and SPS (D'zurilla et al., 2003). There are many studies examining the mediating roles of self-concept and self-esteem in the literature. The mediating role of self-esteem between parenting and adolescents’ social problem-solving skills was examined, and it was found that self-esteem had a positive role in solving social problems when positive parenting (democratic parenting style) were involved while self-esteem had a negative role in solving social problems when negative styles (authoritarian and protective parenting styles) were identified (Kayaalp and Gündüz, 2018).

The studies also provided evidence that self-concept played a mediating role in the relationship between the negative parenting and children’s social behaviors (Wang et al., 2007) and maternal child-rearing style and adolescents’ peer relationships (Deković and Meeus, 1997). Regarding self-esteem, the full or partial mediating role of self-esteem was presented between inappropriate parenting and peer attachment (Lim, 2020), mother attachment and peer attachment (Doğruyol and Yetim, 2019), and parental closeness, monitoring, peer approval, and adolescent aggression (Özdemir et al., 2017). In addition, academic self-concept was found to play a mediating role between negative parenting and prosocial behaviors (Sangawi et al., 2018). In addition, few research results revealed that self-efficacy mediated the relationship between democratic parenting style and academic performance (Masud et al., 2016).

Based on these findings, it is understood that the self-concept plays a mediating role between family characteristics (such as parenting styles, behaviors, relationships, attachment) and the relationships of individuals with their environment (such as social behaviors, peer relations, emotional intelligence, behavioral problems, and prosocial behaviors). Therefore, the findings regarding the mediating role of self-concept support the hypothesis of this study that self-concept plays a mediating role in the relationship between parenting styles and SPS.

### 1.5 The present study

**Figure 1:**
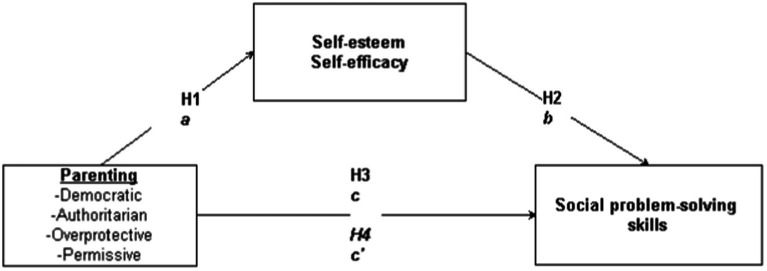
Predicted social problem-solving model.

The studies on the mediating role of self-concept were conducted with adolescents and primary school children (Deković and Meeus, 1997; Özdemir et al., 2017; Sangawi et al., 2018; Wang et al., 2007). However, there is a need for studies with young children in Türkiye that examine parenting styles, SPS, and self-concept together. These studies will provide insights into the mediating role of self-concept and enable comparisons between research results on self-concept in young children (Marsh and Ayotte, 2003) with research results reporting that multidimensional self-concept emerges at a very early age (4–4.5-year-old) (Dapp and Roebers, 2018; Measelle et al., 1998). Moreover, examining the self-esteem and self-efficacy dimensions of self-concept (Bandura, 1993) will contribute to the relevant literature. The impact of parenting on child outcomes can vary depending on the culture being studied (Rudy and Grusec, 2006). Therefore, investigating the links between parenting styles, self-concept, and SPS in young children in Turkish culture, which has both individualistic and collectivist characteristics (Hofstede et al., 2010; Özdemir et al., 2017) may facilitate cultural comparisons. Although, Turkish culture exhibits a structure dominated by collectivist characteristics, it is in a process of transformation in which individualist tendencies are on the rise, especially among younger generations, and the tendency to make individual decisions is on the rise. Türkiye’s transition from a traditional collectivist culture to an individualist culture has accelerated with the dynamics of modernization, and in this process, the levels of individualism and collectivism have differentiated depending on factors such as age, place of residence and social status. While vertical individualism and vertical collectivism have significant effects on social dominance and perceptions of justice, the promotion of individualization by modernization processes has created an area of tension with collectivist traditions. At the same time, a mixed cultural structure in which collectivist values and individualist norms coexist has emerged in Turkish society under the influence of Westernization and modernization, and this transformation has paved the way for the reshaping of social norms (Karataş, 2012; Ünlü et al., 2016). In this context, it is not possible to evaluate parenting styles independent of the cultural context. In every culture, parents apply child-rearing strategies in their daily lives that are appropriate to the needs of that culture in order to transfer their own value system and attitudes to their children (Kağıtçıbaşı, 2007; Maccoby and Martin, 1983). Therefore, the purpose of this study is to examine the mediating role of self-concept in the relationship between parenting styles and SPS in young Turkish children. It is hypothesized that positive parenting styles will lead to a positive self-concept, which in turn will have an impact on young children’s SPS. The research model and hypotheses are presented Figure 1.

*H_1_*: Parenting styles are related to children’s self-esteem and self-efficacy.

*H_2_*: Children’s self-esteem and self-efficacy are related to their SPS.

*H_3_*: Parenting styles are related to children’s SPS.

*H_4_*: Self-esteem and self-efficacy have mediating roles in the relationship between parenting styles and children’s SPS.

## 2 Methods

The present study was designed using a cross-sectional survey model in order to achieve the descriptive and predictive aims of the research (Christensen et al., 2015). This model was chosen because the study aimed to examine the relationships between parenting styles, self-esteem, self-efficacy, and SPS in children aged 48–72 months. The study also aimed to determine the mediating role of self-esteem and self-efficacy in the relationship between parenting styles and the SPS of children.

### 2.1 Participants

The research sample for this study comprised 200 preschool children and their mothers, who were residing in Erzurum, a province located in the eastern region of Türkiye. Of the participating children, 52.5% were aged between 48 and 60 months (*n* = 105, mean age = 56.3, SD = 2.8), while the remaining 47.5% were between 60 and 72 months old (*n* = 95, mean age = 64.7, SD = 3.3). The gender distribution of the children was equal, with 50% males (*n* = 100) and 50% females (*n* = 100). In terms of preschool attendance, 63.5% of the children had attended for 1 year, 24.5% had attended for over a year, and 12% had attended for less than 6 months. Regarding the mothers who participated in the study, 10% had completed primary school (*n* = 10), 5.5% had completed secondary school (n = 11), 25% had graduated from high school (*n* = 50), 19.5% held an associate degree (n = 39), and 40% had completed higher education (*n* = 80). In terms of age, 11% of the mothers were between 18 and 25 years old (*n* = 22), 25% were in the 26–33 age range (n = 50), 47.5% were between 34 and 41 years old (*n* = 95), and 16.5% were in the age range of 42–49 (*n* = 33).

### 2.2 Measures

#### 2.2.1 Social problem-solving (SPS)

Wally Social Problem-Solving Test was created by combining Spivack and Shure (1974) “Preschool Problem-Solving Test” and Rubin and Krasnor’s (1986) “Child Social Problem-Solving Test.” The test was compiled by Carolyn Webster Stratton as part of the “The Incredible Years” project and adapted to Turkish culture by Yilmaz and Tepeli (2013). In the context of the Wally Social Problem-Solving Test, children are shown 15 color pictures describing hypothetical problem situations and asked *what they would do* if they encountered such a problem. The behaviors cited by the child are grouped as prosocial and non-prosocial. Each prosocial behavior is scored “1” point while non-prosocial behaviors are scored “0.” The test consists of separate picture cards for boys and girls. As a result of the validity and reliability studies, the internal consistency reliability coefficient (KR 20) of the items was determined as 0.79. In addition, test–retest reliability was calculated as 0.96.

#### 2.2.2 Parenting styles

Parental Attitude Scale (PAS) was developed by Karabulut-Demir and Şendil (2008) to measure parental behaviors towards children between the ages of 2–6. The 5-point Likert type scale, which can be applied to parents with children between the ages of 2–6, has 4 sub-dimensions and 46 items. The parenting styles assessed in this measure are analyzed in four sub-dimensions: authoritative/democratic (17 items, *α* = 0.94, e.g., “I encourage my child to perform tasks independently.”), authoritarian (11 items, *α* = 0.82, e.g., “I raise my voice to my child when he or she misbehaves.”), overprotective (9 items, *α* = 0.81, e.g., “I protect my child from tasks that might be physically demanding for him or her.”), and permissive (9 items, α = 0.72, e.g., ‘I let my child sleep when he/she wants to.’). The original study was widely used with Turkish samples and reported moderate to high internal consistency values (0.74–0.83). In addition to these values, similar reliability values have been obtained in recent studies using PAS (see Akaroğlu, 2024; Allen and Kara, 2024). On the scale, parents are asked to express how often they display the behavior in question. Scores are calculated separately for each dimension, yielding a score for each. Obtaining a high score on a dimension means adopting the behavior style represented by that dimension.

#### 2.2.3 Self-concept

DeMoulin Self-Concept Developmental Scale developed by Donald DeMoulin between 1995 and 1998, the measuring tool provides both a diagnosis and a systematic and comparative analysis of children’s self-concept. The scale consists of two sub-dimensions as “Self-esteem” (15 items) and “Self-efficacy” (15 items) with a total of 30 items. Afterward, the validity and reliability studies of the scale were repeated for 36–72-month-old children by Zembat et al. (2015), and based on the analysis, Item 15 was removed from the scale. Hence, a structure consisting of two sub-dimensions “Self-esteem” (14 items) and “Self-efficacy” (15 items) was obtained with a total of 29 items. The total Cronbach Alpha value of the scale was 0.810, while the Cronbach Alpha for the “Self-Esteem” subscale was 0.68 and the Cronbach Alpha for the “Self-efficacy” subscale was 0.69. In the assessment form, 3 points are awarded for each smiley face, 2 points for faces with no expression, and 1 point for each unhappy face.

### 2.3 Procedure

#### 2.3.1 Data collection

After the research was designed, ethics committee approval was obtained from the Department of Educational Sciences Ethics Committee of Ataturk University. The study adhered to ethical guidelines by obtaining consent forms from families to ensure voluntary participation, and by seeking permission from teachers and school administrators to obtain the necessary data. Data collection was carried out in schools that approved voluntary participation. Furthermore, measurement tools were not administered to children who expressed their reluctance to participate in the data collection process. The study involved one-on-one sessions with children in suitable, minimally-distracting areas, such as the parent-teacher association room. The researcher was introduced to the children by the classroom teacher and spent time with all of them. Data collection tools, including the Wally Social Problem-Solving Test and the DeMoulin Self-Concept Developmental Scale were administered to each child during 30-min sessions. In addition, parenting styles and personal information were also collected from the mothers of the children in the study group.

#### 2.3.2 Statistical data analyses

SPSS 22 statistical package program was used in data analysis. First of all, the distribution of the data was examined. Secondly in this study, skewness and kurtosis values in the range of −/+2 were taken into consideration (George and Mallery, 2010; Hair et al., 2010; Tabachnick and Fidell, 2007). Subsequently, the third step involved assessing the presence of multicollinearity among the independent variables by examining their tolerance and VIF values. According to Hair et al. (2010), the cases where the tolerance value does not exceed 0.10 and the VIF value does not exceed 10 are acceptable. In this study, tolerance and VIF values obtained from regression models were found below the acceptable threshold values. Finally, the Durbin-Watson coefficient was used to test for autocorrelation and it was found to be below 2 for all models tested. A coefficient below 2 indicates no autocorrelation (Kalaycı, 2010).

PROCESS MACRO software was used in the study to examine the mediating role of self-esteem and self-efficacy in the relationship between parenting styles and SPS PROCESS MACRO is used to calculate path coefficients, standard errors, and t and *p* values of all variables in the tested model by using ordinary least squares (OLS) regression. It also calculates the Bootstrap confidence intervals of the obtained values. The Bootstrap method was used for the mediating effect of self-esteem and self-efficacy. The mediating role of the variables was considered to be statistically significant when the lower limit (BootLLCI) and upper limit (BootULCI) of the Bootstrap analysis results did not include zero values at the 95% confidence interval (Hayes, 2013). Values from mediation analyzes are presented based on mediation typologies specified by Zhao et al. (2010).

## 3 Results

The current study’s results comprise the outcomes of regression analyses that investigated the mediating roles of self-esteem and self-efficacy in the link between parenting styles and SPS. The following section presents the study’s results.

In Table 1, the study found that there were significant correlations between several variables. There was a moderate positive relationship between DA and SPS, and low positive relationships between DA, self-esteem, and self-efficacy. AA and SPS were negatively correlated, while OA and SPS had no significant relationship. Self-esteem was negatively correlated with OA but not self-efficacy. PA was negatively correlated with SPS, self-esteem, and self-efficacy. There were moderate, positive relationships between SPS, self-esteem, and self-efficacy. Gender and age were not found to be associated with the variables. These relationships formed the starting point for testing the hypotheses established in the research.

**Table 1:** Relationships between the variables, and descriptive statistics.

	1	2	3	4	5	6	7	Skew	Kurtosis	M	SD
DA (1)	1							−0.467	−0.492	67.80	10.00
AA (2)	−0.500^**^	1						0.350	−0.508	25.13	7.35
OA (3)	0.228^**^	−0.060	1					−0.291	−0.497	32.70	5.95
PA (4)	−0.266^**^	0.530^**^	0.060	1				0.074	−0.315	21.62	5.17
SPS (5)	0.405^**^	−0.177^*^	−0.084	−0.193^**^	1			−0.421	1.794	9.95	2.60
Self-esteem (6)	0.166^*^	0.027	−0.187^**^	−0.154^*^	0.407^**^	1		−0.353	−1.041	29.82	7.15
Self-efficacy (7)	0.236^**^	−0.032	−0.136	−0.145^*^	0.416^**^	0.867^**^	1	−0.359	−1.363	30.92	9.23

DA, Democratic style; AA, Authoritarian style; OA, Overprotective style; PA, Permissive style; SPS, Social problem solving. *p < 0.05, ** *p* < 0.01.

**Figure 2:**
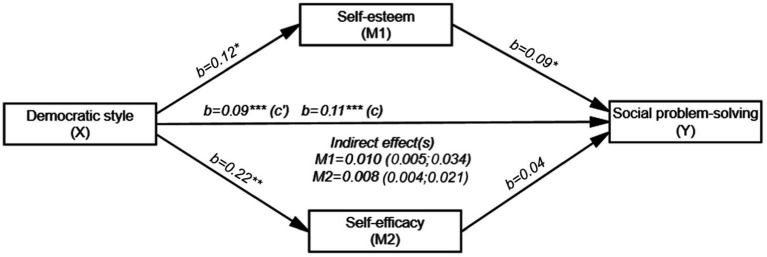
Mediating roles of self-esteem and self-efficacy in the relationship between DA and SPS (*N* = 200). ^*^p < 0.05, ^**^p < 0.01, ^***^p < 0.001; b: Unstandardized beta coefficient; Bootstrap resampling = 5,000.

Figure 2 shows that the overall effect of DA on SPS (path c) was positive and significant (*b* = 0.11, SE = 0.02, t (198) = 6.230, *p* < 0.001 and 95% CI [0.072; 0.138]). The direct effect of DA on self-esteem (path a) (*b* = 0.12, SE = 0.05, t (198) = 2.364, *p* < 0.05 and 95% CI [0.020; 0.217]) and the direct effect of self-esteem on SPS (path b) was found to be positive and significant (*b* = 0.09, SE = 0.04, t (196) = 1.991, *p* < 0.05 and 95% CI [0.001; 0.175]). The direct effect of DA on self-efficacy (path a_i_) was positive and significant (*b* = 0.22, SE = 0.06, t (198) =3.424, *p* < 0.01 and 95% CI [0.093; 0.346]), but the direct effect of self-efficacy on SPS (path bi) was not significant (*b* = 0.04, SE = 0.04, t (196) = 1.991, *p* = 0.302 and 95% CI [−0.032; 0.104]). In addition, the direct effect of DA on SPS (path c’) was also found to be significant (*b* = 0.09, SE = 0.02, t (196) = 5.373, *p* < 0.001 and 95% CI [0.055; 0.119]). DA, self-esteem and self-efficacy together explained 21% (*R*^2^ =0.210) of the variance in the SPS. The indirect effect of DA on SPS was significant. Therefore, self-esteem was the mediating variable in the relationship between DA and SPS (*b* = 0.010; 95% CI [0.001; 0.026]). However, the mediating effect of self-efficacy in the relationship between DA and SPS was not significant (*b* = 0.008; 95% CI [−0.004; 0.021]).

**Figure 3:**
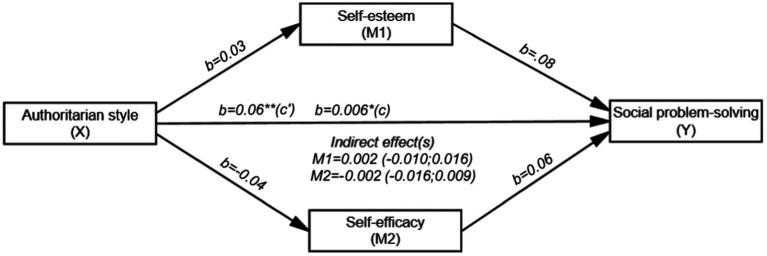
Mediating roles of self-esteem and self-efficacy in the relationship between AA and SPS (*N* = 200). ^*^p < 0.05, ^**^p < 0.01, ^***^p < 0.001; b: Unstandardized beta coefficient; Bootstrap resampling = 5,000.

Figure 3 shows that the overall effect of AA on SPS (path c) was negative and significant (*b* = −0.06, SE = 0.03, t (198) = −2.525, *p* < 0.05 and 95% CI [−0.111; −0.014]). However, the direct effect of AA on self-esteem (path a) (*b* = 0.03, SE = 0.07, t (198) = 0.375, *p* = 0.707 and 95% CI [−0.110; 0.162]) and the direct effect of self-esteem on SPS (path b) were not significant (*b* = 0.08, SE = 0.05, t (196) = 1.777, *p* = 0.077 and 95% CI [−0.009; 0.174]). Similarly, the direct effect of AA on self-efficacy (path a_i_) (*b* = −0.040, SE=0.09, t (198) = −0.450, *p* = 0.653 and 95% CI [−0.217; 0.137]), and the direct effect of self-efficacy on SPS (path bi) were not significant (*b* = 0.06, SE = 0.04, t (196) = 1.668, *p* = 0.097, and 95% CI [−0. 011; 0.130]). However, the direct effect of AA on SPS (path c’) was negative and significant (*b* = −0.06, SE = 0.02, t (196) = −2.756, *p* < 0.01 and 95% CI [−0.107; −0.018]). AA, self-esteem and self-efficacy together explained 15.3% (*R*^2^ = 0.153) of the variance in the SPS. The indirect effect of AA on SPS was not significant. Therefore, the mediating effects of both self-esteem (*b* = 0.002; 95% CI [−0.010; 0.016]) and self-efficacy were not significant (*b* = −0.002; 95% CI [−0.010; 0.016]).

**Figure 4:**
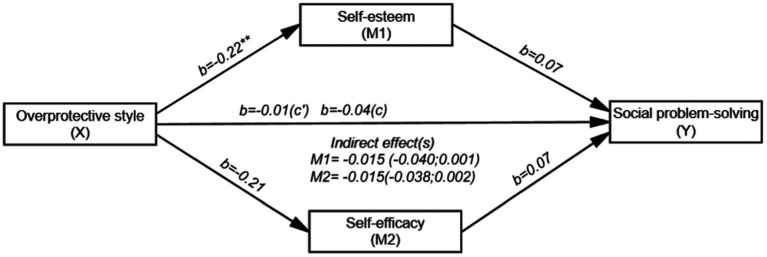
Mediating roles of self-esteem and self-efficacy in the relationship between OA and SPS (*N* = 200). ^*^p < 0.05, ^**^p < 0.01, ^***^p < 0.001; b: Unstandardized beta coefficient; Bootstrap resampling = 5,000.

Figure 4 shows that the overall effect of OA on SPS (path c) was not significant (*b* = −0.04, SE = 0.03, t (198) = −1.186, *p* = 0.236 and 95% CI [−0.098; 0.024]). However, the direct effect of OA on self-esteem (path a) was negative and significant (*b* = −0.22, SE = 0.08, t (198) = −2.675, *p* < 0.01 and 95% CI [−0.390; −0.059]). The direct effect of self-esteem on SPS (path b) was not significant (*b* = 0.07, SE = 0.05, t (196) = 1.412, *p* = 0.159 and 95% CI [−0.027; 0.161]). The direct effect of OA on self-efficacy (path a_i_) (*b* = −0.21, SE = 0.11, t (198) = −1.928, *p* = 0.055 and 95% CI [−0.429; 0.005]), the direct effect of self-efficacy on SPS (path bi) (*b* = 0.07, SE = 0.04, t (196) = 1.956, *p* = 0.051 and 95% CI [−0.001; 0.143]), and the direct effect of OA on SPS (path c’) were not significant (*b* = −0.01, SE = 0.02, t (196) = −0.229, *p* = 0.819 and 95% [−0.063; 0.050]). OA, self-esteem and self-efficacy together explained 12.7% (*R*^2^ = 0.127) of the variance in the SPS. The indirect effect of OA on SPS was not found to be significant. Therefore, the mediating effects of both self-esteem (*b* = −0.015; 95% CI [−0.040; 0.001]) and self-efficacy were not significant (*b* = −0.015; 95% CI [−0.038; 0.002]).

**Figure 5:**
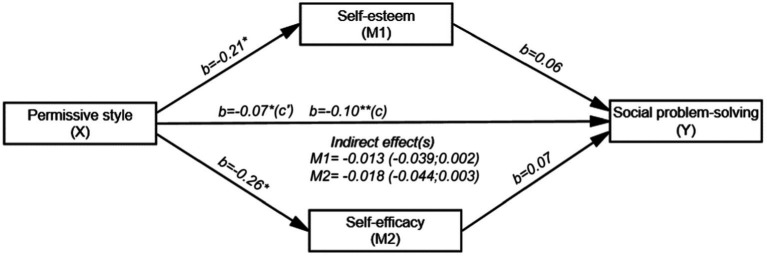
Mediating roles of self-esteem and self-efficacy in the relationship between PA and SPS (*N* = 200). ^*^p < 0.05, ^**^p < 0.01, ^***^p < 0.001; b: Unstandardized beta coefficient; Bootstrap resampling = 5,000.

Figure 5 demonstrates that the overall effect of PA on SPS (path c) was negative and significant (*b* = −0.10, SE = 0.04, t (198) = −2.773, *p* < 0.01 and 95% CI [−0.166; −0.028]). Similarly, the direct effect of PA on self-esteem (path a) was negative and significant (*b* = −0.21, SE = 0.09, t (198) = −2.197, *p* < 0.05 and 95% CI [−0.404; −0.022]). The direct effect of self-esteem on SPS (path b) was not significant (*b* = 0.06, SE = 0.05, t (196) = 1.352, *p* = 0.178 and 95% CI [−0.029; 0.155]). The direct effect of PA on self-efficacy (path a_i_) was found to be negative and significant (*b* = −0.26, SE = 0.12, t (198) = −2.057, *p* < 0.05 and 95% CI [−0.509; −0.011]). However, the direct effect of self-efficacy on SPS (path bi) was not significant (*b* = 0.07, SE = 0.04, t (196) =1.922, *p* = 0.056 and 95% CI [−0.002; 0.140]). The direct effect of PA on SPS (path c’) was negative and significant (*b* = −0.07, SE = 0.03, t (196) = −2.023, *p* < 0.05 and 95% CI [−0.130; −0.002]). PA, self-esteem and self-efficacy together explained 14.3% (*R*^2^ = 0.143) of the variance in the SPS. The indirect effect of PA on SPS was not significant. Therefore, the mediating effects of both self-esteem (*b* = −0.013; 95% CI [−0.039; 0.002]) and self-efficacy were not significant (*b* = −0.018; 95% CI [−0.044; 0.003]).

## 4 Discussion

This study examined the relationships between parenting styles towards young children, self-concept (self-esteem and self-efficacy), and SPS, focusing on the mediating role of self-concept. Initially, the results of the analysis of the relationships between the variables show that parenting styles have significant relationships with each other. While democratic style is negatively related to authoritarian and permissive styles, it is positively related to overprotective style. The authoritarian parenting style has a positive relationship with the permissive parenting style. Examining the relationships between parenting styles, it was found that democratic, authoritarian and overprotective styles produced results consistent with existing literature (Maccoby and Martin, 1983; Tehrani et al., 2024). However, the positive relationship that was identified between the authoritarian and the permissive parenting styles proved to be an interesting finding of this study. This is because the theoretical framework of two orthogonal or unrelated dimensions (i.e., warmth and strictness) proposed by Maccoby and Martin (1983) cannot easily explain the positive correlation between these two styles due to their different characteristics (Chen et al., 2024). Nevertheless, studies conducted in Türkiye using the PAS scale (Allen and Kara, 2024; Akaroğlu, 2024; Karabulut-Demir and Şendil, 2008) have also shown a positive relationship between authoritarian and permissive parenting styles. This finding suggests that parenting styles are influenced by culturally specific features (Darling and Steinberg, 1993; Domènech Rodriguez et al., 2009; Hofstede et al., 2010; Özdemir et al., 2017; Pinquart and Kauser, 2018; Sümer and Kağitçibaşi, 2010). In this context, future research should aim to address these dimensions, as it is essential to consider not only cross-cultural differences but also intra-cultural variations.

When examining the relationships between parenting styles and other variables, it can be seen that the democratic parenting style showed positive significant relationships with SPS and self-efficacy. On the other hand, permissive parenting style showed a negative relationship with SPS and self-esteem. While authoritarian parenting showed a negative relationship with SPS, there was no significant relationship between overprotective parenting and any of the individual variables. Self-esteem and self-efficacy showed strong positive relationships with SPS and with each other. These results suggest that parenting styles have differential relationships with young children’s self-concept and SPS. These relationships are detailed below, along with the mediation results.

The results of the mediation analyses revealed that self-esteem mediated the relationship between democratic style and SPS, whereas self-efficacy did not have a mediating role. The mediating role of self-esteem was found to be an “integrated” type of mediation (Zhao et al., 2010). In this context, it can be argued that functional parenting and high self-esteem help children produce more frequent and positive solutions to social problems in an interactive manner. This finding is consistent with another study conducted with adolescents, where it was found that adolescents’ self-esteem partially mediated the relationship between democratic parenting style and constructive problem-solving. Adolescents who grew up in a family environment with a democratic parenting style had higher self-esteem and solved problems with more effective and constructive strategies (Kayaalp and Gündüz, 2018).

This study provides further evidence for the mediating role of self-concept, specifically self-esteem, in the relationship between parenting styles and children’s social outcomes. This finding is consistent with previous research examining the mediating role of self-concept in the relationship between parenting styles and adolescent peer relationships (Deković and Meeus, 1997). In that study, it was found that positive self-concept and warm, supportive parenting significantly contributed to adolescents’ peer relationships, particularly in the context of high maternal acceptance. Similarly, a study conducted with primary school students reported that positive parenting support for academic self-concept could decrease children’s behavioral problems and increase their prosocial behaviors, which in turn contributed to their social relationships with peers at school (Sangawi et al., 2018). Recent studies have highlighted the bidirectional effect of positive family support on the SPS of young children and adolescents. This bidirectional effect can be interpreted as the family’s indirect contribution to SPS by supporting children’s social skills and self-esteem.

In the current study, it was found that a democratic parenting style had direct and positive effects on children’s self-esteem, self-efficacy, and SPS. This result supports previous research that has shown that a democratic parenting style is most suitable for enhancing SPS, self-esteem, and self-efficacy in young children (Metwally and Akyol, 2018; Şenol and Karaca, 2020). International cross-sectional and meta-analysis studies conducted with samples of children and adolescents have also demonstrated that democratic parenting, which involves parental warmth combined with strictness, enhances SPS (Su et al., 2020) and has positive effects on self-esteem (Jadon and Tripathi, 2017; Martinez et al., 2020; Pinquart and Gerke, 2019), self-efficacy (Yomtov et al., 2015), and self-concept (LeCuyer and Swanson, 2016). Based on the results of this study and in line with previous literature, it can be inferred that the positive effects of democratic parenting style on self-concept and SPS are not contingent upon cultural differences, suggesting that these effects may be universal.

Democratic parents have high acceptance for their children, set manageable goals, and take into account the views of both parents and children in family communication (Baumrind, 1991). They use regulative power, which is clear, direct, rational, and goal-oriented, leading children to use more useful techniques in achieving their goals (Baumrind, 2012). Such children tend to be socially responsible, cooperative, and high in self-regulation, and may effectively solve social problems through interaction and useful techniques (Bloomquist et al., 1996; Su et al., 2020).

Discussions between parents and children during problem-solving provide an opportunity for individualization and autonomy development in children (Vuchinich et al., 1996). Parents’ guidance and modeling transfer to children’s peer relationships. Studies indicate that closeness, intimacy, and cooperation with peers are positively related to SPS (Mize and Cox, 1990). Children’s social information processing and problem-solving skills can be affected by social experiences with parents and peers (Raikes et al., 2013; Raikes and Thompson, 2008). Studies suggest that democratic parenting is negatively associated with children’s behavioral problems, and children with fewer behavioral problems use more effective problem-solving strategies (Eisenberg et al., 2009; Pinquart, 2017; Rudolph and Heller, 1997). Positive parenting, including warmth, sensitivity, high acceptance, and appropriate use of power, positively affects children’s self-control, emotion regulation, and self-regulation skills (Kochanska and Aksan, 1995; Kochanska and Knaack, 2003). Research has also shown that self-regulation is related to SPS (Jelvegar et al., 2014). In this regard, it can be argued that democratic parenting affects children’s SPS both directly and indirectly by reducing problem behaviors and supporting their self-control, emotion regulation, self-regulation skills, and sociability. Based on the results of previous research, it is reasonable to conclude that children with democratic parents use prosocial solution strategies more frequently when solving social problems due to their experiences of cooperation and reconciliation within the family, their parents’ guidance and modeling, and their own self-regulation skills.

According to research, self-esteem plays a more direct and significant role in the development of SPS in children than self-efficacy. In fact, studies have shown that individuals with high self-esteem display positive problem orientation, while those with low self-esteem exhibit avoidance behavior and negative problem orientation (Hamarta, 2009; D'zurilla et al., 2003). This finding is supported by research conducted with older age groups, such as college students and adolescents (D'zurilla et al., 2003; Kayaalp and Gündüz, 2018). Additionally, students with high self-concept tend to be more popular, cooperative, extroverted, and dominant with their peers, as well as displaying positive social interaction skills (Hay et al., 1998). Thus, it can be concluded that self-esteem has a more significant impact on effective and desirable SPS in children, particularly in the early period of their development.

The results of the study on authoritarian parenting style revealed that self-esteem and self-efficacy did not act as mediators in the relationship between authoritarian parenting style and SPS. This implies that self-esteem did not have any positive or negative effect on the strategies used by children of mothers with authoritarian style while solving social problems. This finding contrasts with a previous study conducted in Türkiye with adolescents, which reported that self-esteem played a mediating role between authoritarian parenting style and SPS, and that negative problem-solving approaches were more severe in adolescents with low self-esteem (Kayaalp and Gündüz, 2018). The study also found that the authoritarian parenting style did not have any direct effect on children’s self-esteem and self-efficacy. Therefore, it was concluded that the self-concept of children who grew up with authoritarian parenting style was not negatively impacted by this style. However, the literature provides mixed findings regarding the relationships between authoritarian parenting and self-concept (self-esteem, self-efficacy). While some studies with young children reported similar results to this study (Şenol and Karaca, 2020), other studies have reported that authoritarian style negatively affects self-esteem in young children and adolescents (Kayaalp and Gündüz, 2018; Martinez-Escudero et al., 2020; Pinquart and Gerke, 2019; Tunç and Tezer, 2006), and even affects the self-esteem and self-efficacy of university students in a negative manner (Smith, 2007). A systematic review also suggested that authoritarian parenting has a consistently negative impact on self-esteem, causing damage to children’s self-esteem and increasing feelings of inferiority (Jadon and Tripathi, 2017). Interestingly, the present study suggests that the impact of parenting, particularly in the context of authoritarian parenting, may be influenced by the differences between individualistic and collectivist cultures (Sahithya et al., 2019; Martinez et al., 2020; Rudy and Grusec, 2006), as well as the level of demands placed on children by authoritarian parents (Boer and Tranent, 2013).

Another finding indicate that authoritarian parenting did not directly affect the self-concept of children and their SPS strategies. In other words, the self-concepts of children with authoritarian parents did not influence the strategies they used to solve social problems. However, this result contradicts previous research suggesting a possible negative relationship between SPS and self-concept in children who grew up with authoritarian parents and were negatively affected by this style (D'zurilla et al., 2003; Hamarta, 2009; Kayaalp and Gündüz, 2018).

We concluded that the authoritarian parenting style, which did not affect children’s self-concept, explained SPS directly, and negatively. In other words, children with authoritarian parents preferred more non-prosocial strategies for solving social problems. In this context, this result regarding authoritarian parenting style is compatible with the relevant literature. There are numerous studies examining the links between parenting quality and SPS in the early period and adolescence. In general, these studies determined that authoritarian parenting style such as restraint, low acceptance, aggression, and criticism predicted the use of negative strategies for SPS (Jones et al., 1980; Kayaalp and Gündüz, 2018; Metwally and Akyol, 2018). For example, it was found that children used the avoidance strategy more in solving social problems, could not develop a negotiation strategy, and produced lower-level solution suggestions when the mother’s restraint increased (Dodge et al., 1994; Jones et al., 1980; Kayaalp and Gündüz, 2018; Vuchinich et al., 1996). These findings are consistent with the negative association between authoritarian parenting style and SPS. Authoritarian parents, who tend to use coercive power, focus on hierarchical status in family relations as well as domineering (such as orders and threats) and arbitrary practices. Children have to conform to the expectations and wishes of these types of parents. There is no mutual exchange of ideas in solving a problem. Therefore, children are restrained, suppressed, and forced to comply with the wishes of the adult (Baumrind, 2012). These tactics used by parents are shown to cause children to exhibit behaviors such as resentment, conflict, and avoidance (Baumrind, 1991). In this context, the authoritarian parenting style emerged as a variable that directly affects children’s SPS independent of their self-concept.

Examination of the results regarding overprotective parenting style, it was first concluded that (similar to the authoritarian parenting style) self-esteem and self-efficacy did not have a mediating role in the relationship between this style and SPS. The predictions that the overprotective parenting style negatively affects children’s both self-concept and SPS, and that the low self-concept causes children to select more non-prosocial solutions in solving social problems were not confirmed. No other studies with young children were found regarding these variables. However, a study conducted with adolescents concluded the mediating role of self-esteem between overprotective style and SPS, and the severity of the negative approaches used by adolescents increased while solving social problems (Kayaalp and Gündüz, 2018). Another study determined that dysfunctional parenting (e.g., parental rejection, anxious rearing, and overprotection) had a negative effect on children’s self-esteem and peer relationships. In addition, evidence was presented that these effects explained children’s behavioral problems (Georgiou et al., 2016). However, further studies need to be conducted with young children.

According to the results regarding direct effects, an overprotective parenting style directly and negatively affect self-esteem. It was concluded that the self-esteem of the children who grew up with an overprotective style was negatively affected by this style. Overprotective style was found not to directly affect children’s self-efficacy. The results of a limited number of studies conducted with young children in Türkiye yielded similar evidence to this study (Şenol and Karaca, 2020). In addition, studies with adolescents and university students concluded that overprotection negatively affected self-esteem (Herz and Gullone, 1999; Kayaalp and Gündüz, 2018). Overprotective parents who internally control their children can send the message that they lack security and competence without parental assistance. This style then may harm their self-worth (Laurin et al., 2015), increase their anxiety (Vreeke et al., 2013), and cause children to experience internalization difficulties in the early period (Bayer et al., 2006). Therefore, the self-esteem of the children of parents with overprotective parenting style may have been adversely affected by their low self-worth, perceptions of inadequacy, anxiety, and behavioral problems.

On the other hand, the self-esteem and self-efficacy of children with overprotective parents did not directly affect their SPS. Similar to the results of authoritarian parenting style, the self-concepts of the children of overprotective parents did not affect the strategies they used in solving social problems. This finding in the present research is different from the relevant literature (D'zurilla et al., 2003; Kayaalp and Gündüz, 2018). While it was expected that children whose self-concept was negatively affected by overprotective style would choose strategies that were more dissocial, anxious, or avoidant when solving social problems, the results of the present study were not in line with this prediction. In addition, it was concluded that the overprotective style did not have a total and direct effect on SPS. The results of research examining the relationships between overprotective parenting style and SPS are mixed, similar to the results regarding authoritarian style. Cross-sectional studies conducted in Türkiye reported that overprotective parenting negatively affected young children’s SPS (Kayaalp and Gündüz, 2018; Metwally and Akyol, 2018). It can be argued that this negativity stems from the fact that children with overprotective parents are indecisive, dependent on others (especially parents), insecure, low in self-worth, and anxious (Karabulut-Demir and Şendil, 2008; Laurin et al., 2015). In addition, the results of current research conducted with the young age group also showed that the children of overprotective parents exhibited low sociability and were alienated by their peers (Cooklin et al., 2013; Zhu et al., 2021). However, the fact that overprotective parenting is not associated with children’s SPS may be due to the multidimensional nature of this parenting styles with various implications on children’s adjustment (Power and Hill, 2008), inability to fully clarify parental overprotection, and the differences regarding the antecedents and consequences of such behaviors (Thomasgard and Metz, 1993). Additionally, attention should be paid to both intercultural and intracultural differences in parenting practices (Sahithya et al., 2019).

The study found that self-esteem, self-efficacy, and self-concept did not mediate the relationship between permissive parenting style and SPS strategies in children of permissive parents. The literature did not provide diverse studies for comparison, and the national and international results failed to explain the effect of permissive parenting on children’s self-concept. Furthermore, studies with young children were limited. The study concluded that a permissive style directly and negatively affected children’s self-esteem and self-efficacy. The warmth displayed by permissive parents encourages positive emotions, but the lack of control may hinder children’s development of competence and achievement. Therefore, the effects of these two dimensions should be taken into account when gathering data on permissive parents. One study found no relationship between permissive parenting and young children’s self-concept (Şenol and Karaca, 2020). Studies with adolescents and a meta-analysis showed that permissive parenting positively affected self-esteem (Sharma and Pandey, 2015), while another study found a negative effect on self-concept (Rezai Niaraki and Rahimi, 2013). A recent review reported mixed results on the relationship between permissive parenting and self-esteem (Singh, 2017). Permissive parenting warmth encourages positive emotions, but the lack of control may hinder children’s competence and achievement (Pinquart and Gerke, 2019). In this context, both dimensions should be considered when studying permissive parenting style.

The self-esteem and self-efficacy of children raised by permissive parents did not directly influence their SPS. Similar to the results of authoritarian, overprotective parents, this style of permissive parents did not differentiate children’s self-concept or SPS strategies. This finding in the present study is different from the findings in the relevant literature (D'zurilla et al., 2003; Hamarta, 2009; Kayaalp and Gündüz, 2018). On the other hand, the total and direct effect of permissive style on children’s SPS was negative (Hu and Feng, 2021).

Permissive parenting has been linked to a tendency for children to use non-prosocial strategies when attempting to solve social problems, and some studies have supported a negative relationship between permissive style and young children’s prosocial behaviors, such as cooperation, sharing, helping, and comforting (Hu and Feng, 2021). It is possible that the anxious and atypical behaviors exhibited by children with permissive parents could also contribute to difficulties in solving social problems (Cucu Ciuhan, 2021). However, some studies have found no significant relationship between permissive style and children’s SPS (Metwally and Akyol, 2018). This suggests that other variables not considered in this study may also be impacting the results. Permissive parenting, which is marked by low control and high acceptance, can at times be equated with neglect. Children may have unrestricted access to food, sleep, television, and outdoor activities. Parents who adopt this approach may avoid disciplining their children, shirk their responsibilities, and have minimal expectations for their child’s behavior (Baumrind et al., 2010; Baumrind, 1991; Maccoby and Martin, 1983). Permissive parenting has been linked to several negative outcomes in children. Research has suggested that children with permissive parents may have less autonomy and show immature social–emotional development (Baumrind et al., 2010). Additionally, permissive parenting style has been associated with internalizing and externalizing problems, aggressive behavior, and attachment issues with peers (Rinaldi and Howe, 2012; Llorca et al., 2017). Furthermore, children with permissive parents may have difficulty experiencing and expressing emotions (Wischerth et al., 2016). These findings shed light on the possible reasons behind the results obtained in the current study regarding permissive style. As permissive parenting style is characterized by low control and high acceptance, children raised in such an environment may develop their own ways of solving social dilemmas within the family, without criticism or correction from their parents. This acceptance of the children’s solutions may lead them to believe that their strategies are effective, which could result in the use of similar strategies in other social situations.

### 4.1 Limitations and recommendations

The study had several limitations. First of all, data on parenting styles were obtained only from mothers, not from children. It is known that obtaining parenting data from children or from parents may differentiate the findings (Şenol and Karaca, 2020; Tunç and Tezer, 2006). On the other hand, the lack of data from fathers may have affected the depth of our findings as well (Winsler et al., 2005). In addition, the number of participants was small, and the data lacked observation. It is suggested to support the participation of parents in further research through observation and collecting data from a larger participant sample. Another limitation in the current study was related to examining self-concept on the basis of school sensitivity (self-efficacy) and self-sensitivity (self-esteem) as proposed by Demoulin (1998). In particular, this theoretical basis may have created the prominence of self-esteem in this study and generated self-efficacy findings that did not allow for making effective inferences. It is believed that self-concept data obtained by using different measurement tools can make significant contributions to the literature. Finally, besides the relationship between parenting styles, children’s SPS, and the mediating role of self-concept in these relationships, further studies should investigate all variables in conjunction and address behavioral problems (D'zurilla et al., 2003) which are included in the relevant literature, and which will immensely contribute to the understanding of these relationships.

## 5 Conclusion

The study provides evidence that democratic parenting style may be the most appropriate and culture-independent style for young children’s self-concept, and SPS. The findings of the study indicated that the democratic parenting style supported young children in selecting more effective strategies, and solving social problems, resulting in high self-esteem levels. However, a hierarchical structure was not observed in young children’s self-concept, and self-esteem played an important role in these relationships, rather than self-efficacy. Conversely, the study found no evidence of a mediating effect of either self-esteem or self-efficacy in the association between authoritarian, overprotective, and permissive parenting styles and the SPS of young children. Furthermore, the study did not observe a statistically significant relationship between the self-concept of young children raised under these parenting styles and their SPS.

In addition, the study found that democratic parenting style had a direct positive impact on young children’s SPS, while authoritarian and permissive parenting styles had a negative impact. It was concluded that overprotective parenting style was not associated with SPS. Consequently, the results of this study suggest that democratic parenting style play a crucial role in young children’s SPS, while authoritarian, overprotective, and permissive parenting styles produced mixed relationships. Based on the research findings, it is recommended that parent education programs should be disseminated to promote the positive effects of democratic parenting style on children’s social problem-solving skills (SPS) and self-esteem. These programs can offer guidance to parents to adopt a warm, supportive and disciplined approach. Furthermore, individual counseling and group-based intervention strategies can be developed to reduce the negative effects of authoritarian, permissive and overprotective parenting styles. These interventions should aim to strengthen children’s social skills by improving parent–child interactions.

## Data Availability

The data analyzed in this study is subject to the following licenses/restrictions: data will be shared upon request of the researchers. Requests to access these datasets should be directed to sezer_t@ibu.edu.tr.
